# High Incidence of ACE/PAI-1 in Association to a Spectrum of Other Polymorphic Cardiovascular Genes Involving PBMCs Proinflammatory Cytokines in Hypertensive Hypercholesterolemic Patients: Reversibility with a Combination of ACE Inhibitor and Statin

**DOI:** 10.1371/journal.pone.0127266

**Published:** 2015-05-14

**Authors:** Jeanne d’Arc AlBacha, Mira Khoury, Charbel Mouawad, Katia Haddad, Samar Hamoui, Albert Azar, Ziad Fajloun, Nehman Makdissy

**Affiliations:** 1 Reviva Regenerative Medicine Center, Human Genetic Center, Middle East Institute of Health Hospital, Bsalim, Lebanon; 2 Laboratory of Applied Biotechnology, Azm Center for the Research in Biotechnology and its Applications, Doctoral School for Sciences and Technology, Lebanese University, Tripoli, Lebanon; 3 Department of Biology, Faculty of Science, Section III, Lebanese University, El Kobeh, Lebanon; Baker IDI Heart and Diabetes Institute, AUSTRALIA

## Abstract

Cardiovascular diseases (CVDs) are significantly high in the Lebanese population with the two most predominant forms being atherosclerosis and venous thrombosis. The purpose of our study was to assess the association of a spectrum of CVD related genes and combined state of hypertension hypercholesterolemia (HH) in unrelated Lebanese. Twelve polymorphisms were studied by multiplex PCR and reverse hybridization of DNA from 171 healthy individuals and 144 HH subjects. Two genes were significantly associated with HH: ACE (OR: 9.20, *P*<0.0001) and PAI-1 (OR: 2.29, *P = *0.007), respectively with the occurrence of the risky alleles “Del” and “4G”. The frequencies of the Del and 4G alleles were found to be 0.98 and 0.90 in the HH group *versus* 0.84 and 0.79 in the healthy group, respectively. Serum ACE activity and PAI-I increased significantly with Del/Del and 4G/5G genotypes. The co-expression of Del/4G(+/+) was detected in 113 out of 171 (66.0%) controls and 125 out of 144 (86.8%) HH subjects. Del/4G(-/-) was detected in only 6 (3.5%) controls and undetected in the HH group. Three venous thrombosis related genes [FV(Leiden), MTHFR(A1298C) and FXIII(V34L)] were significantly related to the prominence of the co-expression of Del/4G(+/+). A range of 2 to 8 combined polymorphisms co-expressed per subject where 5 mutations were the most detected. In Del/4G(+/+) subjects, peripheral blood mononuclear cells (PBMCs) produced significant elevated levels of IFN-γ and TNF-α contrary to IL-10, and no variations occurred for IL-4. ACE inhibitor (ramipril) in combination with statin (atorvastatin) and not alone reversed significantly the situation. This first report from Lebanon sheds light on an additional genetic predisposition of a complex spectrum of genes involved in CVD and suggests that the most requested gene FVL by physicians may not be sufficient to diagnose eventual future problems that can occur in the cardiovascular system. Subjects expressing the double mutations (Del/4G) are at high risk for the onset of CVDs.

## Introduction

Cardiovascular diseases (CVDs) encompass all diseases of the heart and blood vessels. They include coronary heart diseases, heart valves diseases, cardiomyopathies, heart rhythm disorders, cerebrovascular diseases and congenital diseases such as heart defects and lower limb arterial disease. CVDs have reached epidemic proportions worldwide and are a critical problem faced by most countries. Today, CVDs are a leading cause of death worldwide [1].

Atherosclerosis is a disease characterized by deposits of fat (mostly cholesterol), cells and other substances in the lining of the arteries of large and medium caliber. This disease causes more morbidity and mortality in the western world than any other disorder. Specific pathways that contribute to endothelial injury or malfunction are not completely understood, but include well-known risk factors such as hypertension, high cholesterol, toxins from cigarette smoke, the homocysteine and hemodynamic factors [2–4]. As a result, atherosclerosis is a chronic and multifactorial disease that progresses slowly over several decades. Hypercholesterolemia (HC) and hypertension (HT) are major risk factors for atherosclerosis, and their coexistence is associated with a yet greater increase in the incidence of cardiac events [5–6]; Hypertension doubles coronary heart disease (CHD) risk. Treating hypertension only reduces CHD risk by about 25%. However, treating hypercholesterolemia in hypertensive patients reduces residual CHD risk more than 35% [6]. Combined influence of environmental and genetic factors can affect hypertension and hypercholesterolmia, and consequently cardiovascular sytem. The development of hypertension is believed to originate from an interaction of lifestyle exposures with multiple genetic factors [7–9]. In addition, in many hypertensive patients, there is strong inherited evidence derived from twin, adoption and family studies indicating that about one third to one-half of the inter-individual variation of blood pressure levels is inheritable [8,10]. The renin-angiotensin system is involved in the formation and progression of atherosclerosis by modulating the vascular endothelial function, the production of proinflammatory cytokines, the proliferation of vascular smooth muscle cells and the synthesis of the vascular matrix [11–12]. The effects of angiotensin II, the product of the angiotensin converting enzyme (ACE) on angiotensin I, on the regulation of vascular tone and blood pressure are mediated by cell surface receptors angiotensin receptor 1 (AGTR1) and angiotensin receptor 2. Given the pathophysiological role of this system and the well established benefits of the ACE inhibitors [13], it has been suggested that genetic variants affecting the function of this system may be considered as genetic risk factors for cardiovascular diseases. A polymorphism resulting from the insertion (Ins)/deletion(Del) of 287 nucleotides in intron 16 of the ACE gene has been extensively studied in relation to the risk of myocardial infarction [14–15]. The Ins/Del polymorphism of the ACE gene and polymorphism by A> C substitution at nucleotide position 1166 of the AGTR1 gene were analyzed in 1162 patients with coronary artery disease (CAD) by S Ye et *al*. [16], suggesting that ACE and AGTR1 gene mutation may contribute to the severity and increased susceptibility to CADs. Niemiec et *al*. came to the same conclusion after analyzing 172 patients with angiographically confirmed premature CAD [17]. A more recent study, in Egypt, also suggests a positive link between ACE polymorphism and premature CAD [18]. Other genes like β-fibrinogen, HPA-1, Apo B and Apo E have also been implicated in atherosclerosis.

Venous thrombosis is the abnormal formation of a blood clot in a vein. Its consequences may vary depending on the location of the clot. From a pathological perspective, plaques with large lipid cores and thin fibrous caps are more prone to rupture, leading to thrombosis and vascular events, as compared to plaques with small and securely contained lipid cores and thick caps [19]. Plasminogen activator inhibitor type-1 (PAI-1) acts as an inhibitor of tissue-type plasminogen activator and urokinase plasminogen activator both of which are responsible for the cleavage of plasminogen into plasmin, an enzyme that regulates fibrinolysis [20]. A 675 amino acid insertion/deletion known as 4G/5G polymorphism has been extensively studied for its relation with venous thrombosis. A meta-analysis suggests that the 4G allele is linked to the increased risk of venous thrombosis, especially when other genetic thrombophilic defects are present [21]. Emerging data also support the role of other mutations in venous thrombosis such as prothrombin, factor V, factor XIII and MTHFR [22]. High prevalence of a mutation at Arg506 in the factor V gene (Leiden, FVL) in patients presenting with venous thromboembolism has emphasized the protective role of activated protein C in neutralizing thrombin, and has highlighted the importance of genetic factors in the pathogenesis of venous thrombosis. FVL is the most common inherited form of thrombophilia; people who have the FVL mutation are at somewhat higher than average risk for a type of clot that forms in large veins in the legs (deep venous thrombosis) or a clot that travels through the bloodstream and lodges in the lungs (pulmonary embolism) [23–25]. Lebanon exhibits one of the highest prevalences of FVL in the world (14.4%) [26]. Nowadays, FVL is the most requested gene by the Lebanese clinicians and it has been considered as marker of predisposition for potential problems that can occur in the cardiovascular system.

In discussing the efficiency or futility of FVL testing, it’s important to note that we observed a dramatic year on year increase in the number of requests for FVL testing in our laboratory service which was accompanied by a concomitant reduction in the number of positive tests (detection of the leiden mutation), suggesting that tests were being requested inappropriately. Thus, despite the presence of recommendations for FVL testing, physicians continue to request the test unselectively. Nevertheless, undetected mutation of FVL is not sufficient to eliminate the risk; additional screening of other genes involved in CVD must be more relevant. Thereby, we conducted a screening analysis of the polymorphisms of 10 CVD related genes and analyzed the eventual combined risk factors.

Extending from the impact of ACE and PAI-1 gene polymorphisms in CVDs, we hypothesized that double mutations of ACE and PAI-1 might increase CVDs susceptibility and severity in the Lebanese population. Thus, it becomes increasingly more important to understand if they coexist or not. Although the prevalence of each risk factor has been studied individually [27,28], the prevalence of the double mutation alone or in association with other atheroscleroris or venous thrombosis related genes has never been investigated neither in healthy nor in hypertensive hypercholesterlomic (HH) patients. Thereby, we conducted a screening analysis of the polymorphisms of these two genes as well as other CVD related genes in 315 Lebanese unrelated individuals (171 controls and 144 hypertensive hypercholesterlemic patients) and analyzed the impact of the “Del” allele and/or “4G” allele in ACE and PAI-1, respectively, on the state (wild-type *versus* mutated) of all selected genes. The effects of ACE inhibitor and statin were studied on the production of pro-inflammatory and anti-inflammatory cytokines from peripheral blood mononuclear cells (PBMCs).

## Materials and Methods

### Subjects

This study extends from 2004 till 2013. Among 1632 outpatients, we selected 315 outpatients divided into two groups: 171 unrelated normotensive normocholesterolemic healthy cases without any family history of CAD or stroke or hypertension or hypercholesterolemia (protocol of selection was approved by an IRB board which restricted the inclusion and exclusion criteria as listed in Table 1) ranging in age from 21 to 78 years defined here as the “Healthy group”, and 144 unrelated hypertensive hypercholesterolemic subjects ranging in age from 21 to 81 years defined here as the “HH group”. All subjects are Lebanese white, and all of their parents and grandparents were born in the same region. Subjects were excluded from the healthy group if they had a history of cardiovascular disease; evidence of cardiac, renal, or cerebral damage or evidence of atherosclerotic plaques at routine clinical and laboratory and echo-doppler examinations. Hypertensive subjects were included if their diastolic blood pressure was superior to 90 mmHg and their systolic blood pressure was superior to 140 mmHg. Hypercholesterolemic subjects were included if their total serum cholesterol was higher than 250 mg/dL. Hypertensive hypercholesterolemic patient with affected parent(s) or grandparent(s) were excluded. Normotensive hypercholesterolemic subjects and hypertensive normocholesterolemic subjects were excluded.

**Table 1:** Characteristics of the study population in the healthy group ^(^ ^1^ ^)^ (results are expressed as the mean ± SEM).

Healthy Group (N = 171)	Male	Female	Mean
Sex	43.8%	56.2%	
Age (years)	41.5 ± 2.1	36.1 ± 3.3	38.8 ± 1.6
Body mass index (kg/m^2^)	24.2 ± 1.1%	22.0 ± 2.8%	
Never smoking	12.1 ± 0.9%	41.9 ± 3.3%	
Ex-smoking	68.4 ± 3.7%	47.2 ± 6.5%	
Current smoker	19.5 ± 4.5%	10.9 ± 4.2%	
Physically active ^(^ ^2^ ^)^	42.0 ± 6.6%	49.8 ± 5.7%	
CAD or Stroke	0	0	
**Exclusion criteria**			
Total Cholesterol (mg/dL)	> 250		
Triglycerides (mg/dL)	> 200		
LDL cholesterol (mg/dL)	> 150		
HDL cholesterol (mg/dL)	< 35		
Fasting glucose (mg/dL)	> 120		
Fasting plasma insulin (μU/mL)	> 10		
Systolic blood pressure	> 140 mm Hg		
Diastolic blood pressure	> 90 mm Hg		
Diabetic	Previously- or currently- treated or untreated		
Stroke	Previously- or currently- treated or untreated		
Body mass index (kg/m^2^)	> 30		

^(1)^ HH group for hypertension and hypercholesterolemics subjects is considered with CAD historical and total cholesterol is > 250 mg/dL proportioned to elevated level of LDL and low HDL, and systolic /diastolic blood pressure is > 140/90 mm Hg.

^(2)^ Physically active: walking or doing other kinds of exercise at least once per week or self-report of moderate/intense level of activity in daily life. LDL: low density lipoprotein; HDL: high density lipoprotein; CAD: coronary artery disease.

### Ethics Statement

All clinical investigations have been conducted according to the principles expressed in the Declaration of Helsinki. All patients provided written informed consent and samples were procured from Reviva Regenerative Medicine Center with review board approval of the research ethics board (REB-MEIH)at the Middle East Institute of Health (MEIH)

### Materials

The CVD StripAssay (VIENNALAB) contains lysis solution, GEN TRACT Resin, amplification Mix of primers, Taq dilution buffer, conjugate solution, sterptavidine-alkaline phosphatase, wash solutions and color developer (Nitro blue tetrazolium (NBT) and 5-bromo-4-chloro-3-indolylphosphatase (BCIP). The supplemented material included the DNA polymerase illustrated rTaq (GE Healthcare). The DNA was extracted from fresh blood samples or blood stored at 4°C, collected on EDTA. Serum was used to test the enzymatic activity of ACE and PAI-1 (Buhlmann laboratories and Invitrogen).

### DNA extraction, multiplex PCR and reverse hybridization

Isolation of genomic DNA was carried out from 315 patients and amplified using biotynilated primers according to the manufacturer’s instructions (Viennalab). Ten genes were amplified and twelve mutations were screened: 5,10 methylenetetrahydrofolate reductase (MTHFR) (C677T and A1298C), β-fibrinogen (FGB) (-455 G>A), angiotensin-converting enzyme (ACE) (I/D (Del/Ins) genotype), apolipoproteins [B (Apo B) (R3500Q) and E (ApoE (E2/E3/E4)) E2/E3/E4 genotype ((codon 112: TGC (Cys)), (codon 112: CGC (Arg)), (codon 158: TGC (Cys)) and (codon 158: CGC (Arg))), blood coagulation factors [II – prothrombin (G20210A), V – leiden (G1691A) and R2 (H1299R), XIII (V34L)], plasminogen activator inhibitor-1 (PAI-1) (4G/5G genotype), platelet glycoprotein IIIa (HPA-1) (L33P (a/b) genotype). The amplification products were hybridized to a test strip containing allele-specific oligonucleotide probes immobilized as an array of parallel lines. Bound biotinylated sequences were detected using streptavidin-alkaline phosphatatse and color substrates.

### Kinetic method of ACE and PAI-1 dosage in serum

The kinetic activity of the ACE enzyme was assessed using the Buhlmann colorimetric kit (KK-ACE). ACE catalyzes the conversion of angiotensin I to angiotensin II. This enzyme also mediates the cleavage of a synthetic substrate, Furylacryloyl-phenylalanyl-glycyl-glycine to glycyl-glycine and furylacryloyl-phenylalanin. The kinetics of this cleavage is measured by observing the decrease in absorbance for 10 minutes at 340 nm. This decrease in absorbance is proportional to the amount of substrate consumed. We used the automated method on all controllers’ clinical chemistry analyzers with at least one open channel. The levels of PAI-1 were mesearued in patient’s serum by ELISA (Invitrogen) following the instructions of the manufactrurer.

### Peripheral blood mononuclear cells preparation, culture and cytokine determination

20 ml of fresh peripheral blood from healthy and HH subjects were collected. Peripheral blood mononuclear cells (PBMCs) were separated from plasma by density gradient centrifugation using Ficoll-Paque PLUS (GE Healthcare). Platelets present in the MNC fraction were discarded by three washes in sterile PBS with 2% FBS. PBMCs were cultured in RPMI 1640 supplemented with 2 mM L-glutamine, 100 U/ml penicillin, 100 μg/ml streptomycin and 10% heat-inactivated FBS. Culture supernatants were harvested after 48 hours and stored at -80°C before the assay. IFN-γ, TNF-α, IL-4, and IL-10 concentrations in culture supernatants of PBMCs were quantified with enzyme-linked immunosorbent assay (ELISA) sets (R&D System, Inc.) as recommended by the manufacturer. The lowest detection limits for IFN-γ, TNF-α, IL-10 and IL-4 were 8 pg/ml, 5.5 pg/ml, 3.9 pg/ml and 10 pg/ml, respectively.

### Statistical analysis

The Statview software (Statview software, SAS Institute, Cary, NC) and Excel 2007 were used for all statistical evaluations. Logistic regression analysis with adjustment for relevant significant variables was used: allelic and genotypic frequencies were compared and statistically analyzed using the Fisher’s exact test, Chi-Square Test and Odds ratio (OR) with 95% confidence intervals (95% CI). The results of kinetic activity of the ACE / PAI-1 are expressed as mean ± SEM and were analysed for statistical significance using Student's t-test. For all statistical tests, *P* values were two-tailed and the level of significance was set at 0.05.

## Results

This study aims at evaluating the prevalence of mutations in genes involved in CVDs in a sample of the Lebanese population. Our study regroups 315 unrelated subjects divided into two groups: “hypertensive hypercholesterolemic” *versus* “healthy” subjects. The Lebanese surveyed women (56.2%) exceed the men (43.8%) and the average combined age of males and females was 38.8±1.6% (Table 1). Twelve gene mutations were screened: ACE(Del/Ins genotype), β-fibrinogen(-455 G>A), HPA-1(L33P(a/b) genotype), ApoB(R3500Q) and ApoE(E2/E3/E4 genotype) mainly involved in atherosclerosis and Prothrombin FII(G20210A), FVL(G1691A),FVR2(H1299R), FXIII(V34L), MTHFR(C677T and A1298C) and PAI-1(4G/5G genotype) mainly involved in venous thrombosis.

### Screening of CVDs related genes in healthy and HH groups

**Fig 1:**
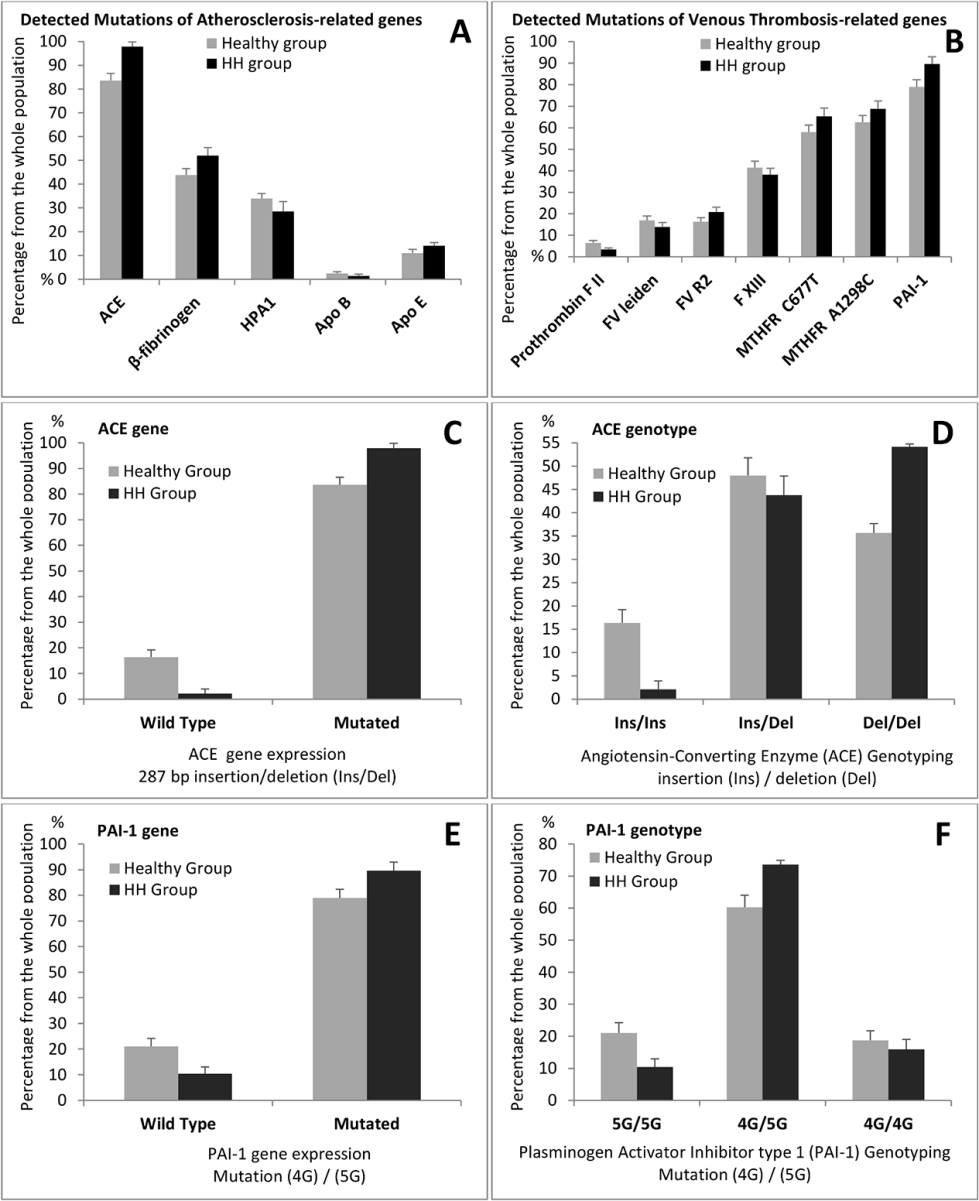
Screening of Atherosclerosis and Venous Thrombosis-related gene’s mutations: allele’s frequencies. DNAs were isolated from peripheral blood and transcribed and amplified by PCR as described in Materials and Methods; DNAs were from hypertensive hypercholesterolemic patients of the HH group (n = 144) *versus* Healthy group (n = 171). Relative distribution of mutated allele of genes related to Atherosclerosis (A) and Venous Thrombosis (B). Frequencies of mutated allele of the Angiotensin-converting Enzyme (ACE) (C) and Plasminogen Activator Inhibitor type 1 (PAI-1) (E) genes *versus* wild type. Polymorphism of ACE gene 287 bp insertion or deletion (Ins/Ins, Ins/Del and Del/Del) (D) and PAI-1 gene (5G/5G, 4G/5G and 4G/4G) (F). Results are expressed as percentages ± SEM of the number of patients detected with or without mutation from the total number of the studied population. Mutations are: ACE(Del), β-fibrinogen(-455G>A), HPA-1(b), ApoB(R3500Q) and ApoE(E4), blood coagulation factors II Prothrombin(G20210A), V(Leiden and R2) and XIII(V34L), the MTHFR(C677T and A1298C) and the PAI-1(4G).

The expression of genes involved in CVDs for the wild-type *versus* the mutated state was evaluated. In the control healthy group and among the atherosclerosis related genes, subjects carrying ACE-Del allele were found 143 out of 171 (83.6%) while the β-fibrinogen (-455G>A) and HPA1 (b allele) were 43.9% and 33.9% of the population, respectively. Apolipoproteins mutations had a lower incidence with 2.3% for Apo B (R3500Q) and 11.1% for Apo E (E4) (Fig 1A). Further investigation of the Apo E gene showed that the E3/3 genotype is the most present (80.8%) followed by E2/3. The frequencies of 7 polymorphisms known to be implicated in thrombosis were: prothrombin FII G20210A (6.4%), FVR2 (16.4%), FVL (17.0%), FXIII V34L (41.5%), MTHFR C677T (57.9%), MTHFR A1298C (62.6%), and PAI-1 4G allele (78.9%) (Fig 1B). In the HH group, the results for the atherosclerosis and venous thrombosis genes combined panels showed similar patterns of mutations’ expression as those of the healthy group with more pronounced profile of results: atherosclerosis genes [ACE (97.9%), β-fibrinogen (52.1%), HPA1 (28.5%), Apo E (14.0%) and Apo B (1.4%) (Fig 1A)] and venous thrombosis genes [FII (3.5%), FVL (13.9%), FVR2 (20.8%), FXIII (38.2%), MTHFR C677T (65.3%), MTHFR A1298C (68.8%), and PAI-1 (89.6%) (Fig 1B)].

### High prevalence of the risky alleles “Del” of ACE and “4G” of PAI-1 genes

The high prevalence of the detected risky alleles “Del” of ACE and “4G” of PAI-1 in the two groups (HH *versus* Healthy) [97.9% *versus* 83.6% (*P*<0.0001) for ACE and 89.6% *versus* 78.9% (*P =* 0.010) for PAI-1, respectively (Fig 1C and 1E)] led us to check the distribution of their genotypes in the studied populations. We observed in the healthy group that the mutated heterozygous genotype “Ins/Del” of the ACE gene is more expressed than the mutated homozygous genotype “Del/Del” as compared to wild type “Ins/Ins” genoptype [Ins/Del (48.0%) > Del/Del (35.7%) > Ins/Ins (16.4%)], whereas the homozygous genotype Del/Del is higher in the HH group [Del/Del (54.2%) > Ins/Del (43.8%) > Ins/Ins (2.1%)] (Fig 1D). For the PAI-1 gene, the heterozygous genotype 4G/5G was significantly higher in both the healthy and HH groups (accounted for 60.2% *versus* 73.6%, respectively) as compared to the mutated homozygous 4G/4G (18.7% *versus* 16.0%, respectively) and to the wild type 5G/5G (21.1% *versus* 10.4%, respectively) (Fig 1F).

ACE (Del) and PAI-1 (4G) mutations were found in equal abundance between males and females in the healthy and HH group: there was no significant difference in the allelic frequency between genotypes of the same group when we compared males to females (data not shown).

### Elevated levels of serum ACE and PAI-1

Next, we measured the kinetic activity of the ACE enzyme in the patients’ serum to evaluate the impact of the “Del” allele. As indicated in Table 2, serum ACE activity was 48.7, 74.0 and 99.0 relative units in Ins/Ins, Ins/Del and Del/Del individuals, respectively in the healthy group and 35.2, 63.5 and 109.2 relative units (respectively) in the HH group indicating that serum ACE activity increases significantly with the Del/Del genotype by about 2.03 fold (*P*<0.05) in the healthy group and 3.1 fold (*P*<0.01) in the HH group.

**Table 2:** Kinetic evaluation of the levels of ACE and PAI-1 in the patient’s serum (results are expressed as the mean ± SEM).

	Healthy group (n = 171)	HH group (n = 144)
**ACE serum level** (relative unit)		
Ins/Ins (I/I)	48.7 ± 6.8	35.2 ± 6.9
Ins/Del (I/D)	74.0 ± 5.6 (151.9%) *	63.5 ± 7.0 (180.4%) *
Del/Del (D/D)	99.0 ± 9.9 (203.3%) ^#^	109.2 ± 7.9 (310.2%) ^# #^
**PAI-1 serum level** (ng/ml)		
5G/5G	10.9 ± 1.3	16.4 ± 1.0
4G/5G	17.8 ± 0.9 (175.2%) ^&^	42.8 ± 1.1 (260.9%) ^&^
4G/4G	20.5 ± 2.2 (202.9%)	44.8 ± 3.4 (273.2%)

*P<0.*05*; I/D *versus* I/I.

^#^ *P<0*.*05*; D/D *versus* I/I.

^# #^ *P<0*.*01*; D/D *versus* I/I.

^&^ *P<0*.*05*; 4G/5G *versus* 5G/5G.

All comparisons were performed within the same group of healthy or HH.

Table 2 shows also the levels of PAI-1 in the serum of the HH group which were elevated comparing to the healthy group. In the case of the 5G/5G genotype, the levels were: 16.4 ng/ml *versus* 10.9 ng/ml (HH *versus* Healthy, respectively). The presence of the 4G allele induced also increases in the levels of secreted PAI-1 in the serum: 1.7 and 2.2 fold of increase in the case of HH group and the increases were 2.6 and 2.7 fold in the case of healthy group (4G/5G and 4G/4G genotypes, respectively).

### Evaluation of the significance relative to the expression of the ACE and PAI-1 genes in the HH and Healthy groups

We compared the group of hypertensive hypercholesterolemic patients with the group of healthy individuals (Total population, n = 315). The odd(s) ratio(s) (OR) were calculated for the 10 genes (12 polymorphisms): mutated allele *versus* wild type (Table 3). Only two genes were significantly associated with the hypertension and hypercholesterolemia (HH): ACE (OR: 9.20, *P*<0.0001) and PAI-1 (OR: 2.29, *P =* 0.007). The significances were related to the homozygous Del/Del genotype in the case of ACE (OR: 11.93, *P*<0.0001), and to the heterozygous 4G/5G genotype in the case of PAI-1 (OR: 2.47, *P =* 0.004). Other polymorphisms involved in atherosclerosis or venous thrombosis were not significantly associated with HH in the studied population.

**Table 3:** Table of Odds Ratios relative to the expression of the ACE and PAI-1 genes in the HH and Healthy groups.

N = 315	HH *versus* Healthy group		
	Odds Ratio	95% CI	*P*
**ACE and PAI-1 Polymorphisms**			
ACE: Del(+) *versus* Del(-)	9.20	2.73 – 30.96	< 0.0001 *
PAI-1: 4G(+) *versus* 4G(-)	2.29	1.19 – 4.39	0.007 *
ACE: Del/Del *versus* Ins/Ins genotype	11.93	3.46 – 41.11	< 0.0001 *
ACE: Del/Ins *versus* Ins/Ins genotype	3.07	1.26 – 7.49	0.007 *
PAI-1 : 4G/4G *versus* 5G/5G genotype	1.73	0.77 – 3.86	0.129
PAI-1 : 4G/5G *versus* 5G/5G genotype	2.47	1.28 – 4.78	0.004 *
**Other Polymorphisms**			
FII (G20210A) *versus* WT ^1^	0.52	0.18–1.54	0.175
FV (Leiden) *versus* WT	0.79	0.43–1.47	0.277
FV (R2) *versus* WT	1.34	0.76–2.38	0.191
FXIII (V34L) *versus* WT	0.87	0.55–1.37	0.314
MTHFR (C677T) *versus* WT	1.37	0.87–2.16	0.110
MTHFR (A1298C) *versus* WT	1.32	0.82–2.10	0.151
FGB (-455G>A) *versus* WT	0.21	0.13–0.36	1.217
HPA-1 (b) *versus* WT	0.78	0.47–1.25	0.180
Apo B (R3500Q) *versus* WT	0.59	0.11–3.26	0.426
Apo E (E4) *versus E3/3*, *E2/2*, *E2/3*	1.29	0.66–2.52	0.280

^1^ WT = Wild Type

### Co-expression of the allele “Del” of ACE and “4G” of PAI-1 (Del/4G,+/+)

We sought to investigate subjects who co-express the mutated alleles (“Del” and “4G”) of ACE and PAI-1 genes. Among the 171 studied cases of the healthy group, we found only 6 cases (3.5%) not expressing neither the “Del” nor the “4G” alleles (absence Del/4G(-/-)); however, the co-expression of Del/4G(+/+) was detected in 113 out of 171 subjects (66.1%), and the expression of only one of the alleles “Del” or “4G” was detected in 52 subjects. Among the 144 studied cases of the HH group, we didn’t find any subject not expressing neither the “Del” nor “4G” alleles (absence Del/4G(-/-) (0%)); however, the co-expression of Del/4G(+/+) was detected in 125 out of 144 subjects (86.8%), and the expression of only one of the alleles “Del” or “4G” was detected in 19 cases (Table 4). In fact, the co-expression of Del/4G(+/+) was significantly associated with HH (OR: 14.37, *P =* 0.051).

**Table 4:** Co-expression of ACE/PAI-1 genes polymorphism Del/4G.

	Number of patients expressing or not the double risk factors “Del/4G”	
	Del/4G (-/-)	Del/4G (+/+)
**HH group** (n = 144)	0 (0%)	125 (86.8%)
**Healthy group** (n = 171)	6 (3.5%)	113 (66.0%)

Odds ratio: 14.37; 95% CI: 0.80–258.04; *P*-value: 0.051

**Fig 2:**
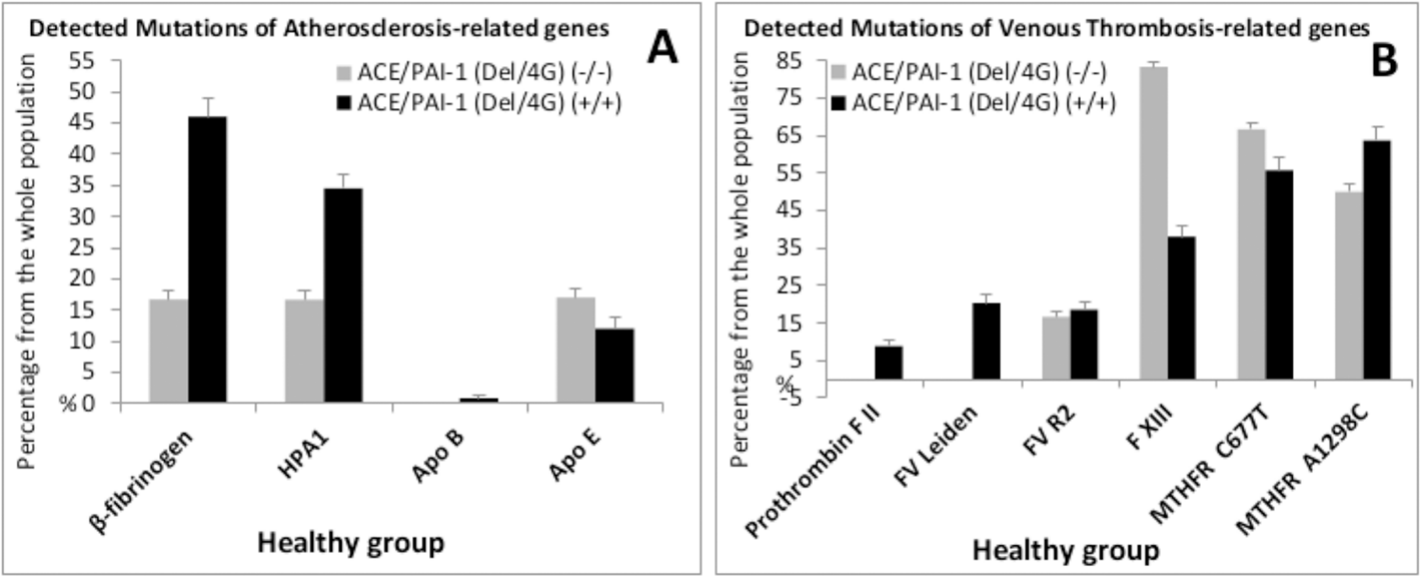
Distribution of atherosclerosis and venous thrombosis-related genes in the healthy group (n = 171) between populations co-expressing allele “Del” of ACE and “4G” of PAI-1(Del/4G,+/+): Allele’s frequencies. *A*, Relative distribution of atherosclerosis related polymorphisms. *B*, Relative distribution of venous thrombosis related genes. Results are expressed as percentages ± SEM of the number of patients detected with or without mutation from the number of the studied population. Co-expression Del/4G(+/+) (detected in 113 patients) or absence of Del/4G(-/-) (only 6 patients not expressing alleles Del/4G) (Total population, n = 171).

Second, we were interested to check whether the other CVDs related polymorphisms were influenced or not by this co-expression Del/4G(+/+) of the ACE and PAI-1 genes. The absence of subjects that didn’t express the Del and 4G alleles (Del/4G(-/-)) in the HH group didn’t allow any comparison between the HH group and the Healthy group. Therefore, we evaluated the expression of a spectrum of 10 polymorphisms that could be influenced by the co-expression Del/4G(+/+) among the group of healthy individuals (Fig 2). Among the atheroscelrosis related genes (Fig 2A), the variations of the expression of FGB, HPA1, ApoB and ApoE did not reach the significance (Table 5) and were: 16.7%, 16.7%, 0%, 17.0% (Del/4G(-/-)) *versus* 46.0%, 34.5%, 0.9%, 12.0% (Del/4G(+/+)), respectively. Among the venous thrombosis related genes (Fig 2B), FV(Leiden), MTHFR(A1298C) and FXIII(V34L) were significantly related to the prominence of the co-expression of the mutated alleles Del/4G(+/+) [(0%, 50.0%, 83.3%, *versus* 20.4%, 63.7%, 38.1%) (Del/4G(-/-) *versus* (+/+)), respectively]. In fact, FVL and MTHFR(A1298C) increased [(OR: 2.4, *P* = 0.007) and (OR: 2.78, *P* = 0.015), respectively] contrary to FXIII(V34L) which decreased [(OR: 0.033; *P =* 0.005)] (Table 5). The variations reflected the heterozygous state of FVL (0% *versus* 15.9%), the homozygous states of MTHFR(A1298C) (0% *versus* 16.8%) and FXIII Val34Leu (50.0 versus 6.2%), respectively for Del/4G(-/-) *versus* Del/4G(+/+).

**Table 5:** Table of Odds Ratios relative to the expression of 10 polymorphisms genes in the groups co-expressing or not the alleles Del/4G of the ACE/PAI-1 genes.

N = 119	Del/4G (+/+) *versus* Del/4G (-/-)					
	Heterozygous			Homozygous		
	Odds Ratio	95% CI	*P*	Odds Ratio	95% CI	*P*
FII (G20210A)	1.83	0.22–15.02	0.480	2.03	0.24–16.50	0.430
FV (Leiden)	2.40	0.13–44.91	0.007 *	0.66	0.03–13.69	0.351
FV (R2)	1.24	0.43–3.58	0.430	2.07	0.20–20.70	0.450
FXIII (V34L)	0.25	0.02–2.93	0.271	0.033	0.003–0.36	0.005 *
MTHFR (C677T)	0.43	0.08–2.46	0.290	1.6	0.07–37.00	0.057
MTHFR (A1298C)	1.29	0.24–6.74	0.541	2.78	0.13–58.29	0.015 *
FGB (-455G>A)	3.77	0.42–33.38	0.203	0.98	0.04–20.27	0.469
HPA-1 (b)	2.57	0.28–22.76	0.350	1.67	0.73–3.85	0.149
Apo B	0.58	0.06–5.77	0.510	0.38	0.03–4.40	0.420
Apo E (E4)	2.63	0.32–21.2	0.309	0.77	0.08–7.07	0.589

### Evaluation of the number of risk factors to occur by patient

**Fig 3:**
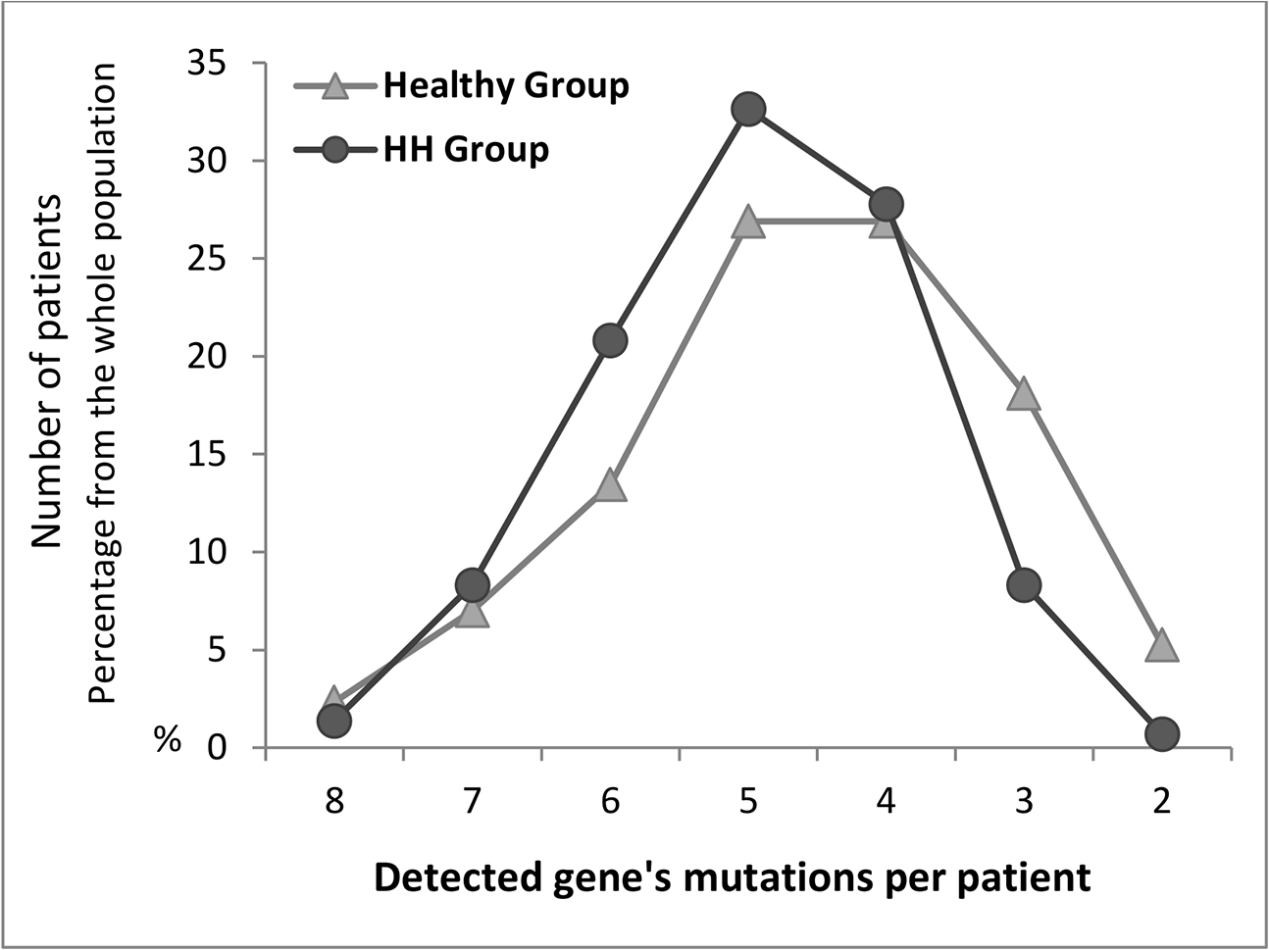
Distribution of risk factors that occur by gene’s mutations. Co-expression of mutations by patient detected in total population of the healthy group (n = 171) or the hypertensive hypercholesterolemic group (n = 144). Results are expressed as percentages of from the total number of the studied population.

**Fig 4:**
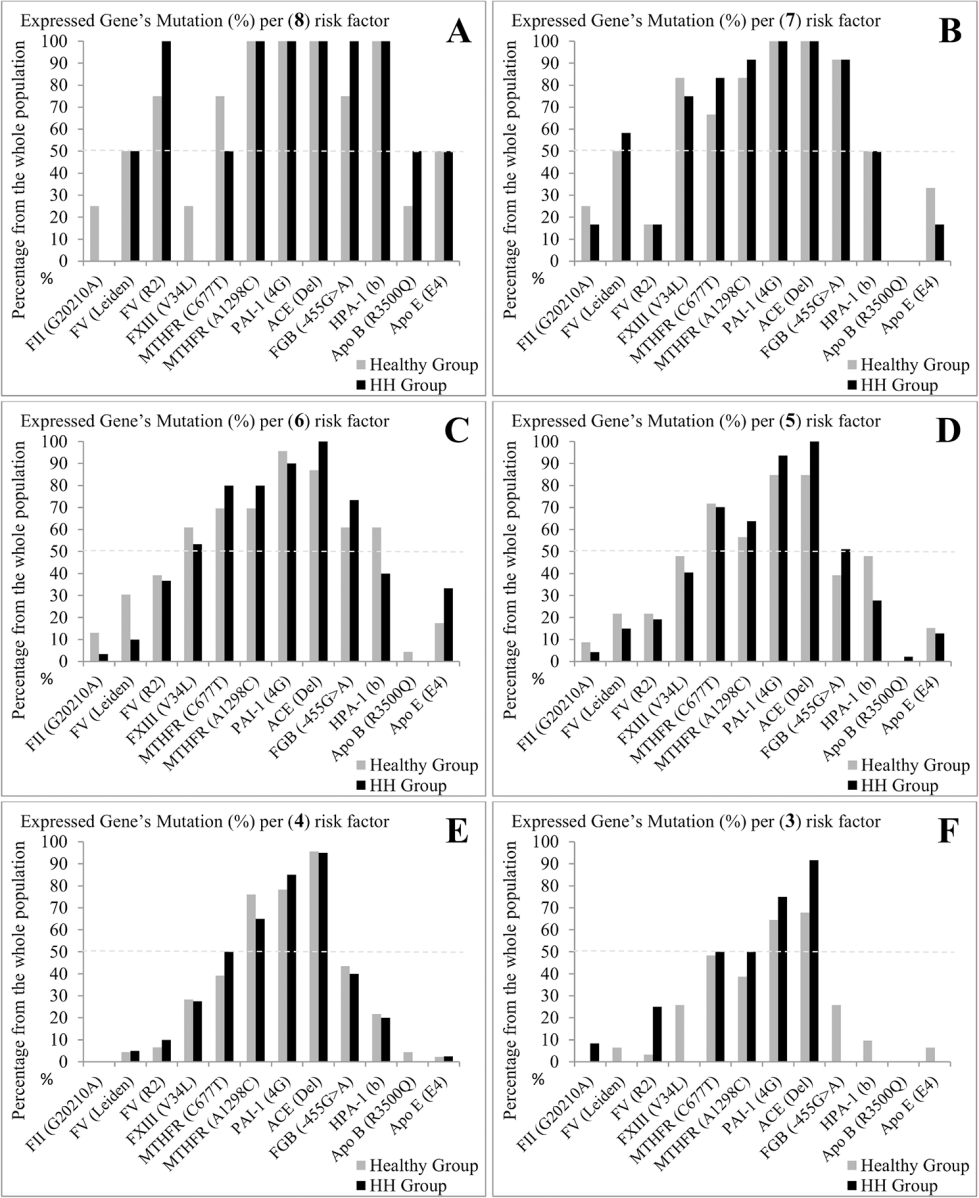
Evaluation of the number of risk factor to occur by patient between the Healthy and the HH groups. Results are expressed as percentages of expressed gene’s mutation per (n) risk factor: 8(*A*), 7(*B*), 6(*C*), 5(*D*), 4(*E*) and 3(*F*). 12 polymorphisms are studied: Prothrombin FII(G20210A); FV-L(Leiden); FV-R2(R2); FXIII(V34L); MTHFR(C677T); MTHFR(A1298C); PAI-1(4G); ACE(Del); beta-fibrinogen FGB(-455G>A); HPA-1(b); ApoB(R3500Q); ApoE(E4).

Among the healthy group, 5.3% of the population expressed a minimum of 2 mutations and 2.3% of the population expressed a maximum of 8 combined mutations per subject, with a higher prevalence of 26.9% for a detection of 4 and 5 mutations per subject (Table 6). In the HH group, 32.6% of the population expressed 5 mutations (Fig 3). The comparison between the healthy and the HH groups showed that 27–33% of the studied population co-expressed at least 4 to 5 mutations with dominance of the HH group. As indicated in Fig 4, the most expressed combined mutations were for the genes: ACE, PAI-1, MTHFR, FXIII and β-fibrinogen with predominance of ACE(Del) and PAI-1(4G).

**Table 6:** Evaluation of the number of risk factors to occur by subject.

Combined risk factors per subject (n)	Number of subject expressing (n) risk factors	
	Healthy group (171 subjects)	HH group (144 subjects)
**8** ^(^ ^1^ ^)^	4 (2.3%)	2 (1.4%)
**7**	12 (7.0%)	12 (8.3%)
**6**	23 (13.5%)	30 (20.8%)
**5**	46 (26.9%)	47 (32.6%)
**4**	46 (26.9%)	40 (27.8%)
**3**	31 (18.1%)	12 (8.3%)
**2**	9 (5.3%)	1 (0.7%)

(1) One risk factor is defined as a detected mutation per gene expression

### Production of pro-inflammatory cytokines in subjects co-expressing Del/4G(+/+)

**Fig 5:**
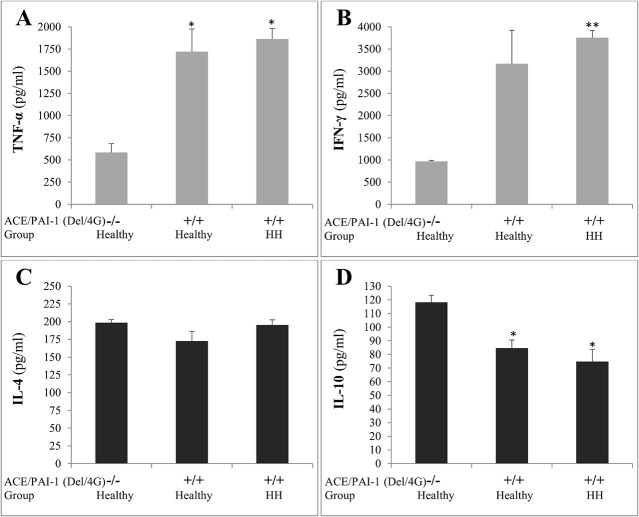
Profile of cytokines secretion in cases co-expressing or not ACE/PAI-1(Del/4G). Cytokines secretion in culture supernatants from peripheral blood mononuclear cells (PBMCs) obtained from the 6 healthy subjects not expressing the combined double risky alleles “Del” of ACE and “4G” of PAI-1 genes [Healthy-ACE/PAI-1(Del/4G)(-/-)], from 6 healthy subjects co-expressing Del/4G [Healthy-ACE/PAI-1(Del/4G)(+/+)] and from 6 patients HH co-expressing Del/4G [HH-ACE/PAI-1(Del/4G)(+/+)]. TNF-α (A), IFN-γ (B), IL-4 (C) and IL-10 (D) production were determined in triplicates (n = 24 for each group) and results are Mean ± SEM. *P<0.*05* and **P<0.*01*; Del/4G(+/+) *versus* Del/4G(-/-).

We finally investigated the ability of cells expressing Del/4G(+/+) to produce the pro-inflammatory cytokines TNF-α and IFN-γ, and the anti-inflammatory cytokines IL-4 and IL-10 in comparison to controls Del/4G(-/-). The PBMCs of the 6 healthy subjects found to be Del/4G(-/-) were used as controls to two groups each of 6 subjects co-expressing Del/4G(+/+) selected from the healthy and HH groups. Carriers of the double mutations produced significantly high levels of TNF-α and IFN-γ, and low levels of IL-10 (Fig 5). However, the production of IL-4 was not significantly affected.

**Fig 6:**
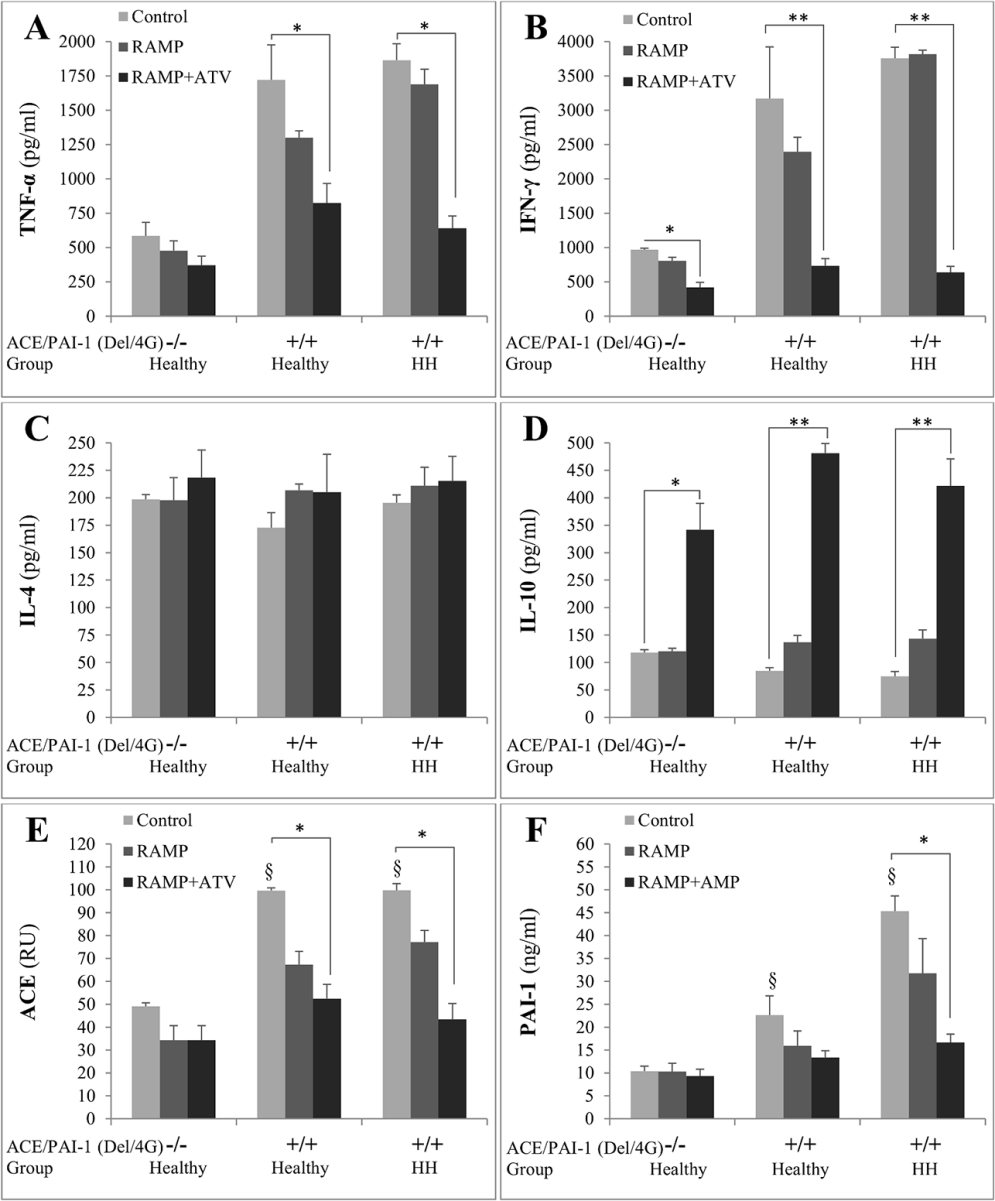
Effects of ramipril (RAMP) and atorvastatin (ATV) on cytokines, ACE and PAI-1 secretion. Culture supernatants from peripheral blood mononuclear cells (PBMCs) were obtained from the 6 healthy subjects not expressing the combined double risky alleles “Del” of ACE and “4G” of PAI-1 genes [Healthy-ACE/PAI-1(Del/4G)(-/-)], from 6 healthy subjects co-expressing Del/4G [Healthy-ACE/PAI-1(Del/4G)(+/+)] and from 6 patients HH co-expressing Del/4G [HH-ACE/PAI-1(Del/4G)(+/+)]. Cells were treated 48h with 10μM of RAMP and/or ATV. TNF-α (A), IFN-γ (B), IL-4 (C), IL-10 (D), ACE (E), PAI-1 (F) production were determined in triplicates (n = 24 for each group) and results are Mean ± SEM. ^§^ *P<0*.*05*; Del/4G(+/+) *versus* Del/4G(-/-).*P<0.*05* and **P<0.*01*; RAMP+ATV *versus* Control.

It has been reported that ACE inhibitors have beneficial effects on hypertension and statins on hyperlipidemia. Therefore, in this study, we examined the effects of ACE inhibitor (Ramipril (RAMP)) in combination or not with statin (Atorvastatin (ATV)) on the production of cytokines (Fig 6). Ramipril alone did not reverse the situation observed in Del/4G(+/+) whatever the cases healthy or HH patients. However, treatments of PBMCs with RAMP+ATV reversed significantly the situation: decreases in the levels of TNF-α (maximum effect -66%, Fig 6A) and IFN-γ (maximum effect -83%, Fig 6B), and increases in the levels of IL-10 (maximum effect 5.7 fold, Fig 6D). The levels of IL-4 weren’t affected by the treatment (Fig 6C).

In Del/4G(+/+) PBMCs, the levels of ACE and PAI-1 produced in the supernatants increased significantly with the presence of the double mutations (2.02 and 2.03 fold for ACE; 2.2 and 4.3 fold for PAI-1) (Healthy and HH, respectively) (Fig 6E and 6F). RAMP alone reduced the levels of ACE and PAI-1 in the supernatants of PBMCs, but the reductions did not reach the significances. Nevertheless, RAMP+ATV significantly reversed the increases respectively for ACE and PAI-1: -47% and -41% in the case of healthy-Del/4G(+/+), and -57% and -63% in the case of HH-Del/4G(+/+), considering then that the combined treatment may promote reversibility of the effect produced by the co-expression of the alleles Del and 4G of ACE and PAI-1 genes.

## Discussion

In this study, we examined the polymorphisms of 10 genes and their 12 mutations implicated mainly in atherosclerosis and venous thrombosis. The spectrum of the different detected mutations co-expressed by patients was analyzed. Whole blood was collected from (1) 171 unrelated healthy individuals, normotensive and normocholesterolemic; (2) 144 unrelated patients, hypertensive and hypercholesterolemic and was tested as described in the methods part for the mutations of the selected genes. The results show that ACE and PAI-1 genes are significantly associated with the hypertension and hypercholesterolemia, and clearly illustrate a spectrum of five polymorphisms highly detected in the two groups: ACE, PAI-1, MTHFR, FXIII and β-fibrinogen. Among the 315 studied cases, subjects carrying a combined status of 2 polymorphisms ACE(Del) allele and PAI-1(4G) allele were found 238 out of 315 (75.6%) (Del/4G(+/+)) subjects and only 6 out of 315 (1.9%) (Del/4G(-/-)) subjects. More interestingly, among the 144 subjects of the HH group, 125 subjects (86.8%) were Del/4G(+/+); however, Del/4G(-/-) was undetectebale (0%). In addition, FV(Leiden), MTHFR(A1298C) were significantly associated with the double expression Del/4G(+/+), contrary to the FXIII(Val34Leu) which showed significant decrease in its expression. Pro-inflammatory cytokines (TNF-α and IFN-γ) were produced by PBMCs derived from subjects expressing the double mutations, contrary to IL-10.

Among the atherosclerosis-related genes, ACE gene showed in the two studied populations the highest incidence of mutation. Our results are in total concordance with a previously published study on 133 Lebanese [27]. ACE serum kinetic was highest in Del/Del individuals, a result that confirms previous study [29]. The frequency of the Del allele in the HH group was significantly higher (97.7%) as compared to the Ins/Ins (2.1%) with predominance of the homozygous genotype Del/Del (54.2%). These data indicate that ACE Del allele might be more prevalent within a hypertensive hypercholesterolemia population. Our results showed also that the mutated allele’s frequencies of the beta-fibrinogen and HPA-1, two other atherosclerosis-related genes, were elevated. The correlation between ACE and the two genes has not been studied in the Lebanese population and no published data could be found about the correlation; however, the frequencies of the mutated alleles of beta-fibrinogen and HPA-1 have been previously reported in separate studies [30,31]. Here, we found that these two genes were not significantly associated with the Del allele of ACE gene. Among the other atherosclerosis-related genes regardless of the studied group healthy or HH, we found ApoE3/3 genotype was the most prevalent in our study: the prevalence of ApoE genotypes E3/3, E3/4, and E2/3 was found to be 81%, 10%, and 7%, respectively, and 0–1% for each of E2/4, E4/4 and E2/2 genotypes in the healthy group; very close results were found in the HH group: 79% (E3/3), 14%(E3/4), 7%(E2/3), and 0% (E2/4, E4/4 and E2/2). The Lebanese population studied here showed similarities to earlier reported ApoE genotypic distributions (high E3 allele frequency and total absence of E2 allele) [32]. Furthermore, although meta- analyses suggest that apoE4 carriers may have a 40–50% increased CVD risk [33]; a recent publication showed that 77.4% out of 53 Turkish individuals expressed the apoE3/3 genotype and suggests that the apoE3 is associated with more severe disease than E2 allele [34]. Finally, the R3500Q mutation of the ApoB gene was not observed in the general Lebanese population and our results are consistent with a previous work [35].

Among the venous thrombosis-related genes, MTHFR and PAI-1 were the two genes with the two highest percentage of mutation: MTHFR [C677T (65.3% vs. 57.9%) and A1298C (68.8% vs. 62.6%)], and PAI-1 (89.6% vs. 78.9%) (HH vs. Healthy, respectively groups). The frequencies of the mutated alleles of FXIII(V34L), FV(R2 and Leiden) and prothrombin (G20210A) were less detected: respectively 41.5%, 16.4%, 17.0% and 6.4% in the healthy group compared to 38.2%, 20.8%, 13.9% and 3.5% in the HH group. Here, we also suggest that the association of both factors may increase the risk of CVDs considering the individual role of each of them in venous thrombosis.

Wild-type PAI-1 (5G/5G) gene was present in the Healthy and the HH groups (21.1% vs. 10.4%, respectively) while the heterozygous genotype (4G/5G) was expressed in 60.2% and 73.6% of the population and the homozygous genotype (4G/4G) in 18.7% and 16.0%, respectively. Our results on the PAI-1 distribution were a bit different than those obtained by Shammaa et al. [28] (45.6%, 36.9% and 17.5% for 4G/5G, 5G/5G and 4G/4G, respectively). The difference between our study and the other studies could be explained by the very wide heterogeneity of the Lebanese population [36] or by the selection of the studied populations.

Concerning the MTHFR gene, Sabbagh et al [37] previously showed that 50.2% of 205 tested Lebanese healthy individuals carried the A1298C mutation of the MTHFR gene; our data showed that this mutation is more frequent. Our results showed also higher frequencies in the two studied groups but concur with the previous findings on other populations, and are consistent with others reports who stated the co-occurrence of MTHFR677CT and 1298AC genotypes associated with increased risk of hypertension [38–40]. Recently, genetic polymorphisms related to the MTHFR gene are associated with the risk of hypertension particularly when accompanied with obesity and diabetes among Saudi subjects [41]. More recently, a meta-analysis from 114 Studies with 15411 cases and 21970 controls suggested that the MTHFR C677T polymorphism may be associated with hypertension and hypertension in pregnancy, especially among East Asians and Caucasians [42]. The 677C/T MTHFR polymorphism was previously associated with essential hypertension but also with coronary artery disease and higher homocysteine levels [43]. MTHFR polymorphism has been also reported to accelerate CAD in familial hypercholesterolemia [44].

Concerning the other thromogenic factors, DNA from 205 unrelated healthy Lebanese donors previously showed that the prevalence (25.8%) of V34L carriers of the FXIII gene differed from our study (41.5%) [45]. FVR2 and Leiden were less detected in our populations [46,26], but this cannot eliminate their implications in CVDs; FVR2 plays a role as a risk factor for venous thrombosis in mutated patients through an increased thrombin generation [47]. FVL is the most common inherited form of thrombophilia [23–25]. Lebanon exhibits one of the highest prevalences of FVL in the world (14.4%) [26]. Nowadays, FVL is the most requested gene by the Lebanese clinicians and it has been considered as marker of predisposition for potential problems that can occur in the cardiovascular system. FVL is a routine test though its utility in routine testing is still very much debatable [48]. However, its role in venous tromboembolism (VTE) had been well documented. In a Lebanese group of 68 VTE patients, 69 CAD patients and 192 randomly selected healthy subjects [49], while the prevalence of FVL in CAD patients was not statistically different from that of healthy subjects, the authors reported a significant increase (70.6%) in FVL prevalence which was seen in VTE patients, strongly implicating the involvment of FVL in the onset of VTE. At our knowledge, there are no published studies on the onset understanding of the CVDs that investigated a regrouped panel of thrombogenic factors. However, Goodman et al [50] reported in a studied panel of a thrombogenic gene mutations that PAI-1 4G/5G, factor XIII V34L, and homozygous MTHFR C667T correlated significantly with recurrent pregnancy loss.

Hypertension is a well-known prevalent disorder among Lebanese population especially in regions characteristically having high frequency of consanguinity plus a high aggregation rate of familial diseases as hypertension, obesity, and diabetes. Tohme et al [51] revealed in a sample of 2125 Lebanese adults from all regions in Lebanon and proportionate with the respective population density that 23.1% of the respondents admitted being hypertensive. Despite this fact, so far little data has been published concerning the genetic background of Lebanese subjects in terms of their susceptibility to hypertension. The first study conducted on the renin-angiotensin system (RAS) gene polymorphisms in 124 Lebanese hypertensive patients demonstrated a possible association of the AGT T and AT(1)R C alleles with hypertension but not the ACE Del/Del polymorphisms although the Del allele frequency was high (77%) in the tested patients [52]. In our study, the two polymorphisms Del/Del and Ins/Del were significantly associated with the HH, where the frequency of the Del allele was elevated (97.9%).

Concerning the hypercholesterolemia factor, previous reports confirmed the higher prevalence of familial hypercholesterolemia (FH) in Lebanon [53] and showed a relationship with other mutations in the LDLR gene especially combined heterozygosity which can cause a severe phenotype [54]. Several studies have established that the renin-angiotensin system, including the ACE insertion/deletion genetic polymorphisms has been implicated in the pathogenesis of essential hypertension [7,55]. A high prevalence of the Del allele has been shown in hypertensive and dyslipidemia subjects throughout the world [56–65]. AlHarbi et al [66] investigated the association of ACE Ins/Del polymorphism and FH in Saudi population. In their study, FH individuals tended to have higher Del/Del (48.4%) genotypes and more Ins/Del (32.9%) genotypes than controls. However, there was no significant difference in the allelic frequency between the FH patients and controls. O’Malley et al. [67] showed that in heterozygote FH patients with ACE Del/Del genotype, the incidence of myocardial infarction is 2.5 times higher and coronary heart disease is 2.2 higher than in those with Del/Ins or Ins/Ins genotypes. Survival rate of carriers with ACE Del/Del genotype was reported to be significantly lower than that of Ins/Del and Ins/Ins carriers [68]. Zhang et al [69] clearly showed, in a group of 155 patients with CAD, that major adverse cardiovascular events (MACE) occurred more frequent in patients with the Del/Del genotype of the ACE gene. In fact, the increased activity of ACE by the Del allele led to the increased production of angiotensin II and enhanced the degradation of the cardiovascular protective agent bradykinin. This resulted in the hypertrophy of the muscle and caused the instability of the atherosclerosis plaque, and was subsequently followed by unstable angina or MI. In the same study [69], the prevalence of 4G/4G genotype was significantly higher in the MACE patients, and 4G/4G genotype was more frequent in multiple vessel disease than in single vessel disease.

Our data clearly showed that a combined state of double mutations Del/4G of the ACE/PAI-1 genes is highly detected. Among the 171 studied cases of the healthy group, only 6 patients (3.5%) did not express neither the “Del” nor the “4G” alleles Del/4G(-/-); However, Del/4G(-/-) was undetectebale in the HH group of 144 patients. This indicates a potential involvement between these genes and the increased HH risk. In fact, the increased ACE activity caused by Del/Del polymorphism may play an important role in elevating the plasma levels of PAI-1, thus contributing then to the balance of the fibrinolytic pathway [70]. Maurizio Maragaglione and other authors reported that cells with 4G/4G genotypes carrying the highest circulating levels of PAI-1[71–73]. Zhang et al [74] reported that essential hypertension patients have elevated serum ACE and plasma PAI-1 levels, and the increased ACE level was due to the Del/Del polymorphism which may play an important role in elevating plasma PAI-1 level. The genetic variation of ACE contributes to the balance of fibrinolytic pathway, which may be one of the pathological mechanisms linking the ACE genotype and essential hypertension. On the other hand, the 4G/4G genotype of the PAI-1 gene was previously reported to be significantly associated with high cholesterol [75]. The expression of PAI-1 mRNA in aorta was reported to be significantly increased when vascular injury was combined with hyperlipidemia. A marked increase in PAI-1 gene expression was seen when arterial injury was accompanied by hypercholesterolemia and the authors suggested that increased synthesis and stabilization of vascular PAI-1 may potentiate accumulation of extracellular matrix, thereby accelerating atherosclerosis [76]. Out of a set of 37 CVD-associated genetic markers and a set of 17 thrombogenic, inflammatory, and metabolic blood markers, Corsetti et al [77] showed that the 4G/5G polymorphism of the PAI-1 gene was the only polymorphism to be associated with the risk for recurrent coronary events in a subgroup of normolipidemic postinfarction patients. Loew et al [78] reported that the co-existence of the 4G/5G polymorphism of the PAI-1 gene and the Ins/Del polymorphism of the ACE gene increases the risk for early onset of coronary heart disease, more espacially with the Del/Del polymorphism of ACE before the age of 55 years after controlling for sex, age, smoking, diabetes, hypertension, hyperlipidemia and school education. Our data suggest that the high incidence of the co-expression of ACE(Del) and PAI-1(4G) are at high risk for the onset of CVDs.

Contrary to the FVL and MTHFR(C677T /A1298C) which were significantly associated with the double expression Del/4G(+/+), the FXIII Val34Leu showed significant decrease in its expression. In fact, plasma factor XIII (FXIII) or “fibrin stabilizing factor” is an enzyme of the blood coagulation system considered as the precursor of a transglutaminase that cross-links fibrin, thereby altering its properties, including resistance to fibrinolysis. Alterations in factor XIII activity could affect thrombosis risk. There are 4 common polymorphic forms of FXIII that differ among ethnic groups. The Val34Leu polymorphism results in an amino acid change near the thrombin cleavage site that may alter the rate of activation. Several case-control studies have investigated the relation between the Val34Leu polymorphism and venous thromboembolism. Some have shown a potentially protective effect of this polymorphism, but the association is not consistent. Evidence is conflicting regarding the association of the factor XIII Val34Leu polymorphism with risk of venous thromboembolism [79], it is noteworthy that Kohler et al. [80] described higher concentrations of PAI-1 and an increased frequency of the PAI-1 4G/4G genotype in patients with the FXIII 34Leu genotype and myocardial infarction (MI), suggesting that impaired fibrinolysis negates the postulated protective effect of the FXIII 34Leu genotype. The blood coagulation factor XIII, which covalently cross-links fibrin by catalyzing the introduction of γ-glutamyl-ε-lysine peptide bonds between fibrin γ- and α-chains [81], and PAI-1, which plays a central role in controlling the fibrinolytic system. Homozygosity for the deletion genotype (4G/4G) has been associated with PAI-1 concentrations higher than those associated with the insertion genotype (5G/5G), and hence with reduced fibrinolytic activity [82,71]. The association between factor XIII-A (FXIII-A) Val34Leu polymorphism and MI risk remained controversial. Chen et al [83] suggested in their meta-analysis that FXIIIA Val34Leu polymorphism was a protective factor for MI in caucasians. Gohil et al [84] described among 126525 VTE cases and 184068 controls derived from 173 case-control studies, which included 21 genes (28 polymorphisms), FVL, Prothrombin, PAI-1 4G/5G, ACE Del/Del were found to be significantly associated with VTE contrary to FXIII Val34Leu showed significantly protective effects. Here we show that the Val34Leu polymorphysim in the healthy group is decreased when patients express either the Del allele of ACE or the 4G allele of PAI-1 alone and it’s less present in the group of patients co-expressing Del/4G(+/+) compared to Del/4G(-/-) (38.1% vs. 83.6%, respectively): mutated homozygote [6.2% vs. 50.3%, Del/4G(+/+) vs. (-/-), respectively] and heterozygote [31.9% vs. 33.3%, Del/4G(-/-) vs. (+/+), respectively]. These data show evidently that the homozygous mutation is the factor that may be influenced by the co-expression of the alleles Del and 4G of ACE and PAI-1. The diminution of the expression of the homozygous state of the mutation when Del/4G are co-expressed, probably indicates a decrease in the protective role it may play and this may correlate with increasing the risk of CVDs. Furthermore, it is important to note that in the HH group where there was no patients not expressing the double mutation (Del/4G,-/-) and 125 patients expressing Del/4G(+/+), the frequency of the Val34Leu polymorphism of the FXIII was detected as following: mutated homozygote (7.2%) and heterozygote (31.2%). These data of the HH group are closely similar to that observed with the Healthy group (6.2% homozygous, 31.9% heterozygous) among the group of patients expressing the double mutation, this indicates that hypertension and hyperchoesterolemia correlates to the expression of this double mutation of the ACE and PAI-1 genes.

Our data show clearly that a pro-inflammatory role may reflects the co-expression of the risky alleles Del and 4G of ACE and PAI-1 genes. TNF-α and IFN-γ increased contrary to the anti-inflammatory IL-10 cytokine. Ramipril alone did not reverse these situations; however, the addition of atorvastatin seems to play a key role. An increasing number of genetic studies and also epidemiological, have proven evidence for a relation between the atherosclerotic cardiovascular disease and chronic inflammatory diseases [85]. By promoting monocyte activation, as well as expression of adhesion molecules by influencing collagen synthesis, tissue factor, and matrix metalloproteinases, IFN-γ and TNF-α can mediate proatherogenic processes [86,87]. Wang et al. [88] showed recently in human peripheral blood lymphocytes derived from normal subjects that atorvastatin significantly decreased the expression of IL-1, IL-6, IL-8, TGF-β1, TGF-β2 and PAI-1. In addition, other numerous studies suggest inhibitory effects of statins on pro-inflammatory cytokine production, such as TNF-α, IFN-γ in mononuclear cells in atherogenesis [89,90]. The increases observed in the case of IL-10 after treatment with RAMP+ATV support the hypothesis of involvement of ACE/PAI-1(Del/4G) in an anti-inflammatory mechanism suggesting that atorvastatin can synergise with ramipril with respect to immunomodulation of cytokines. Atorvastatin, when given to hypercholesterolemic patients, has been found to decrease PBMC production of IFN-γ [91], TNF- α, IL-1 and IL-6 [92] *in vitro*.

In conclusion, in the studied Lebanese population, the incidence of ACE and PAI-1 mutations, separately or together is much higher than that of other genes and the presence of mutations in either one of the two genes or both genes, increase the prevalence of other CVD related genes. These data, together with a long list of published articles showing that the co-mutation of these 2 genes may affect other disorders, suggest that screening for ACE/PAI-1 mutations may be considered as an early potential marker for the onset of CVDs in the Lebanese population but further evaluation consisting of a bigger sample and a follow up of individuals is needed to confirm the real value and possible applications of this screening. Similarly, the study of the environmental impact on subjects with the previous gene polymorphisms can also have a significant importance as of how it may modulate the expression of these potential risk factors [93].
